# Recreational behaviour, risk perceptions, and protective practices against ticks: a cross-sectional comparative study before and during the lockdown enforced by the COVID-19 pandemic in Romania

**DOI:** 10.1186/s13071-021-04944-7

**Published:** 2021-08-23

**Authors:** Silvia-Diana Borșan, Sabina Ramona Trif, Andrei Daniel Mihalca

**Affiliations:** 1 Department of Parasitology and Parasitic Diseases, University of Agricultural Sciences and Veterinary Medicine of Cluj-Napoca, 3-5 Mănăștur Street, 400372 Cluj-Napoca, Romania; 2 Department of Psychology, “Babeş-Bolyai” University, 37 Republicii Street, 4300015 Cluj-Napoca, Romania

**Keywords:** Ticks, COVID-19 lockdown, Questionnaire, Recreational behaviour, Risk perceptions, Protective practices

## Abstract

**Background:**

Gaining insight into the risk perceptions and the knowledge evolution of the public about emerging or changing health risks is vital for the improvement of health promotion activities. Currently, scientific evidence regarding the attitudes of the Romanian public towards ticks is scanty. This study aimed to identify how the lockdown enforced by the COVID-19 pandemic in Romania impacted the recreational behaviour, risk perceptions, and protective practices of the Romanian population regarding ticks.

**Methods:**

A cross-sectional, nationwide web-based questionnaire was designed and distributed via social media to evaluate if, and how, the COVID-19 lockdown impacted the behaviour of the Romanian public concerning ticks. The survey was available online from 6 May until 15 May 2020, which marked the last day of the travel ban in Romania. The collected data were processed by applying both uni- and multivariate methods.

**Results:**

Respondents reported a higher frequency of finding ticks on themselves and their dogs during the lockdown. Bathing/showering and checking the body for ticks were the two most used protective behaviours both before and during the lockdown. Nevertheless, an overall lower usage rate of protective measures was registered during the lockdown. Almost all dog owners used a form of ectoparasite control for their dogs, and only three stopped due to lockdown-associated reasons. Respondent characteristics that were found to be positively associated with risk perceptions were being female and living in peri-urban/suburban/rural environments.

**Conclusions:**

Despite spending less time outdoors during the lockdown, more respondents reported finding ticks on themselves or their dogs. Changes in the preferences for recreational locations, rates of protective practices usage, amount of time spent in specific areas, or tick seasonal activity might have contributed to this outcome. Concerning risk groups, men of all ages, senior citizens, and rural inhabitants should be targeted by the relevant Romanian authorities when promoting local or nationwide tick awareness campaigns.

**Graphical abstract:**

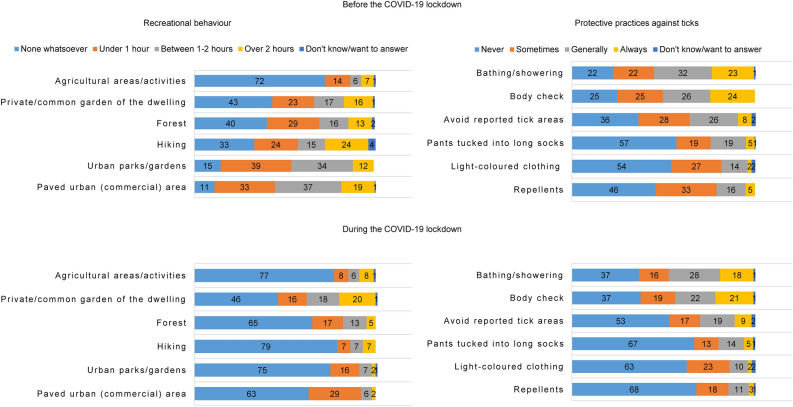

**Supplementary Information:**

The online version contains supplementary material available at 10.1186/s13071-021-04944-7.

## Background

Ticks are vectors of a plethora of zoonotic pathogens that cause diseases in humans and animals, and are thus an increasing focus of attention worldwide. To date, the distribution area of these arthropods, in particular *Ixodes ricinus*, the most widespread questing tick species in Europe, has expanded significantly, comprising northward latitudes, higher altitudes, and a variety of urban and peri-urban habitats. Consequently, tick-borne infections are a growing global public health concern [1].

Lyme borreliosis (LB), the infection caused by the *Borrelia burgdorferi* sensu lato (s.l.) complex is the most common zoonotic tick-transmitted disease in Europe and North America [2]. Other tick-borne pathogens (TBPs) vectored by *Ixodes* spp. such as bacteria of the order Rickettsiales, protozoans of the *Babesia* genus, or *Bartonella* spp., or the tick-borne encephalitis virus (TBEV) can lead to human infections, and in some cases even severe long-term or permanent sequelae [3].

In Romania, 532 human Lyme disease cases were serologically diagnosed and detailed in the most recent annual report of communicable diseases [4]. Concerning the Rickettsiales order, *Anaplasma phagocytophilum* has yet to be described from humans, nonetheless, spotted fever group rickettsiae have been reported from human patients [5, 6]. Given the passive surveillance system of TBEV implemented in 2008 in Romania, the risk of humans contracting this disease is still unknown since there is no regular screening [7]. Nonetheless, variable seroprevalence rates against TBEV in humans and animals across several counties in Romania are reported by Ionescu et al. [8], and Salat et al. [9]. Regarding the TBPs in Romanian dogs, *A. phagocytophilum* and *Babesia canis* were most commonly detected in serum samples tested by Mircean et al. [10], and Andersson et al. [11] by serology. Altogether, data regarding the tick-borne diseases (TBDs) of humans and dogs in Romania is currently limited and outdated.

Gaining insight into the risk perceptions and the knowledge evolution of the public to emerging or changing health risks is vital for the improvement of public health strategies [12–14]. Even though risk perceptions positively influence protective behaviour against various health risks, there are usually discrepancies between the perceived and the objective risk [15]. This is especially true for emerging health risks seen as difficult to manage by laypeople. Such an example is the exposure to prospective infected ticks during the novel coronavirus disease-2019 (COVID-19) pandemic, where mass media, personal networks, and other social instruments may have negatively contributed to the overall perceived risks [16].

The severe acute respiratory syndrome coronavirus 2 (SARS-CoV-2) was first detected in Wuhan, China, in December 2019. Since then, SARS-CoV-2 has spread rapidly, reaching epidemic proportions worldwide, until declared a pandemic on 11 March 2020 by the World Health Organization (WHO) [17]. To prevent the spread of the SARS-CoV-2 in Romania, the Ministry of Internal Affairs declared a national state of emergency by Military Ordinance 1 of 17 March 2020 [18]. Therefore, certain socio-economic activities were either suspended or restricted in terms of the number of participants or their movement at a national or international level. Moreover, from 24 March until 15 May 2020, by Military Ordinance 3, Article 1, the Ministry of Internal Affairs ordered the prohibition of movement for all citizens outside the home/household, with some exceptions [19].

This questionnaire-based survey aimed to assess the impact of the movement restrictions generated by the COVID-19 pandemic on the recreational behaviour, risk perceptions, and protective practices concerning ticks of the Romanian public. Currently, scientific evidence of the aforementioned perceptions in the Romanian population is lacking, and to the best of our knowledge, this is the first study that seeks to close this gap; thus the results herein might prove useful for the development of national public health strategies against tick-borne infections.

## Methods

### Study design

**Fig. 1:**
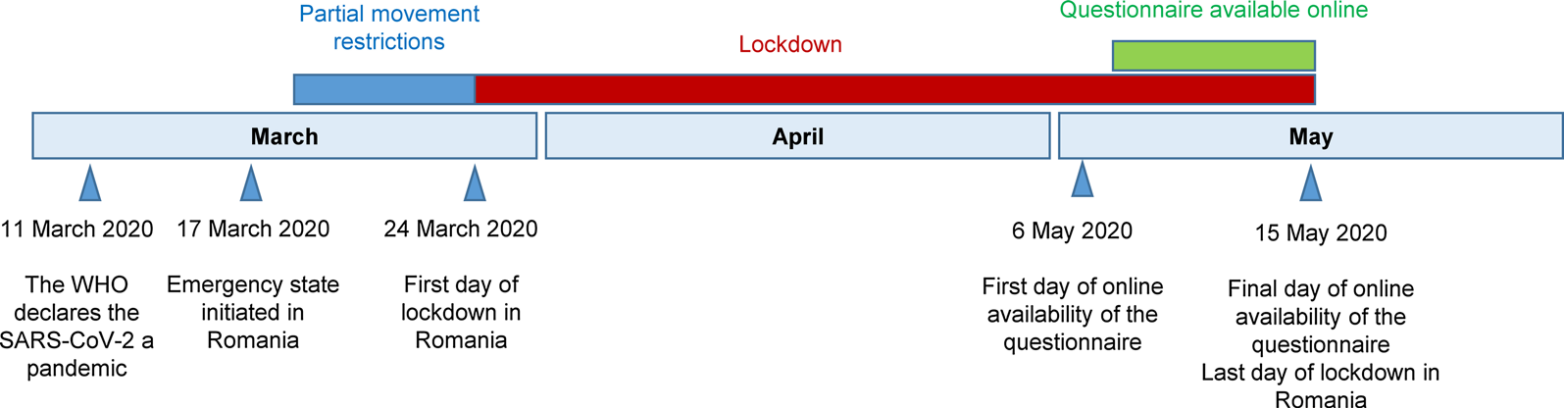
The timeline of the movement restrictions related to the COVID-19 pandemic in Romania and online availability of the questionnaire during 2020

As part of a large-scale investigation into various aspects regarding the ecology of ticks and TBPs in urban areas in Romania ("Multidisciplinary One Health excellence research platform for neglected and emerging vector-borne diseases"), we surveyed the impact the lockdown imposed by the Romanian Government during the COVID-19 pandemic had on the general public concerning potential tick exposure. The descriptive study was cross-sectional and nationwide in scope, and consisted of a web-based questionnaire. The questionnaire was administered to the public using the Facebook platform, precisely by using Facebook groups targeting each county (*n* = 41) in Romania, shared on the business page of the Department of Parasitology and Parasitic Diseases Cluj-Napoca, and personal Facebook pages. A short briefing on the questionnaire was broadcast by TVR1 Channel on national television. Given the means of dispensing, the overall reach of the questionnaire (including the number of people who visualised the form, but chose not to participate) could not be recorded, hence we were not able to assess the response rate. Only respondents aged 18 and above and residing in Romania were eligible for the study. The questionnaire was available online from 6 May until 15 May 2020, which marked the last day of the travel ban imposed by the Romanian Government (Fig. 1).

### Questionnaire

The survey instrument comprised 14 general questions and an additional subset of up to 10 questions, depending on the respondent’s answers. The questions included the following categories: demographic characteristics (age, gender, location/county of residence, child/children in care); characteristics of the residence location; recreational behaviour; self-assessment of risk related to tick bites; and protective practices used against ticks. The questions were designed following a “before/after” pattern, aiming to evaluate the impact of the movement restrictions generated by the COVID-19 pandemic on the general population (Additional file 1: Text S1).

The recreational behaviour of respondents and dog owners was observed using eleven questions. First, we assessed whether the respondents changed their residence as a result of the lockdown. Second, we aimed to register whether the amount of outdoor time for the respondent, its child/children, and dog(s) changed (increased/decreased/no change). Then, by using a set of two questions, the respondents were asked to provide details regarding their preferences for spending free time (amount of time and environment type), before and during the movement restrictions. We also assessed the number of times participants discovered ticks on themselves in the previous year (spring 2019) and during the current study year (2020), before and during the lockdown. Dog owners were questioned about their preferences (amount of time and environment type) for walking their dogs, and the presence of ticks on their dogs before and during the movement restrictions.

Risk perceptions were elicited using three questions where respondents were asked to indicate their opinion on how the movement restrictions imposed by the COVID-19 pandemic impacted the risk of them, their child/children, and/or dog(s), contracting a tick or a TBD.

Finally, two questions in the survey analysed the protective practices used by the respondents against tick bites. To avoid repetition, we assumed the same practices used by the respondent would apply to the child/children in care. Six options of protective methods/equipment were available for the respondents to choose from based on the frequency of usage of each one (i.e. never, sometimes, generally, always). Three questions assessed the protective practices used by dog owners for their dogs. Dog owners were also provided with a list of ectoparasite control methods and asked to choose the appropriate option for their case.

### Data collection

Considering the time restrictions (only 10-day online availability of the questionnaire), our goal was to record as many submissions as possible, without striving for representation in terms of age groups, gender, or county of residence. Thus, setting targets regarding the number of participants based on the demography of the county or country was beyond the scope of the present study. The share of the population with internet access was 75.7% in Romania during 2019, with 61.8% of users located in urban areas [20], implying that web-based surveys might exclude a substantial share of the population residing especially in rural areas in Romania. The data collection was enabled by the JotForm Inc. survey company.

### Data analysis

The collected data was processed using SPSS version 20 software. We excluded from this analysis the “I don’t know/I don’t want to answer” responses concerning the frequency of ticks reports from humans and dogs, and the risk assessment questions. The share (%) or the number of respondents that chose to answer as such are mentioned in the corresponding results section. We first tested the differences between the frequency of tick bites reported before and during the lockdown using a paired-samples *t*-test. To test whether there were differences regarding the frequency of tick bites from the respondents and dog owners concerning demographic data and recreational behaviour, we used ordinary least squares (OLS) regression analysis, with categorical variables analysed with dummy coding. The following variables were tested to see whether they predicted the frequency of tick reports from humans and dogs: gender, age group (< 45 years; > 45 years), change in time spent outdoors, owning a dog (only for ticks reported from humans), residence type, change in residence, recreational preferences, and protective practices (only for ticks reported for dogs). Chi-square analysis was performed to assess the impact of demographic variables (age group, gender, and residence) on the risk perceptions of participants. In cases where the assumptions for the Chi-square were not met (more than 20% of the expected count were less than 5), an extension of Fisher’s exact test (Fisher-Freeman-Halton exact test) was used. Further,* t*-tests were performed to assess the differences in protective measures taken against ticks before and during the quarantine. Finally, to test predictions among demographic data (age group and gender) and the frequency of using various protective measures against ticks, we used multivariate analysis of variance (MANOVA) tests. *P*-values ≤ 0.05 were considered statistically significant. In all cases, asterisks indicate statistical significance: *P ≤ 0.05, **P ≤ 0.01, ***P ≤ 0.001.

## Results

### General characteristics of the study participants

Overall, of the 401 respondents who completed the questionnaire, 74% were female, and 51% belonged to the 25–34-year-old group. Regarding the geographical distribution of the respondents, we registered submissions from 34 out of the 41 total counties in Romania, with the majority of answers collected from Macroregion 1 (North-West and Central Romania). The top three most common residence types reported were urban apartments (45%), followed by peri-urban/suburban apartments (areas bordering cities) (20%), and suburban houses (12.7%). Moreover, 31% of participants reported taking care of a child/children under 18 years old, and 57% owning a dog when completing the survey. Only 9.5% of the participants changed their residence as a direct result of the movement restrictions. Of these, 3.5% switched from urban to rural locations, another 3.5% changed their address but not the environment type, 2% moved from urban to suburban areas, and a few (0.5%) from suburban/rural to urban locations (Table 1) (Additional file 2: Dataset S1).

**Table. 1:** Socio-demographic characteristics of study respondents (*n* = 401)

	*N*	%
Gender		
M	106	26
F	295	74
Age group (years)		
18–24	67	17
25–34	204	51
35–44	76	19
45–54	34	8.5
55–64	12	3
65–74	5	1
75–84	2	0.5
> 85	1	0
NUTS1		
RO1	284	71
RO2	45	11
RO3	53	13
RO4	19	5
Child/children under 18 years old		
Yes	123	31
No	278	69
Owning dog(s)		
Yes	230	57
No	171	43
Residence type		
Urban apartment	181	45
Peri-urban/suburban apartment surrounded by forests/trees/shrub with private/common garden	48	12
Peri-urban/suburban apartment surrounded by a small number of trees and shrubs and no garden	32	8
Urban house with garden/or surrounded by a small number of trees and shrubs	37	9
Suburban house with private/shared garden	44	11
Suburban house without a garden but with access to forest	7	2
House in rural area	47	12
Farm	5	1
Change of residence due to COVID-19 lockdown		
Yes	38	9.5
No	363	90.5

F, female; M, male; T, total; NUTS1, Nomenclature of Territorial Units for Statistics (macroregions). Romania is divided into four NUTS1 as follows: RO1, Macroregion 1 (North-West and Central); RO2, Macroregion 2 (North-East and South-East), RO3, Macroregion 3 (South-Muntenia and Bucharest-Ilfov), RO4, Macroregion 4 (South-West Oltenia and West)

### Recreational behaviour

#### Frequency of reported tick bites in humans

Of the 401 respondents, 13.5% (*n* = 54) reported tick bites during 2020, and one person refrained from answering (Table 2). Overall, more people reported being bitten by ticks during the lockdown (*t*_(399)_ = −3.910, *P* = 0.000). The regression model was significant *F*_(13, 380)_ = 4.828, *P* = 0.000, with an *R*^2^ = 0.142, and the regression coefficients can be observed in (Table 3). The only statistically significant differences concerning the frequency of tick bites recorded are the increase in time spent outside (OLS regression, *B* = 0.207, *SE* = 0.076, *P* = 0.006), hiking (OLS regression, *B* = 0.060, *SE* = 0.020, *P* = 0.004), and time spent in agricultural areas (OLS regression, *B* = 0.044, *SE* = 0.019, *P* = 0.022) (Table 3).

**Table 2:** Frequency of finding ticks on study respondents before and during the COVID-19 lockdown by selected socio-demographic characteristics

Variable *N* (%)		One time *N* (%)		Two times *N* (%)		More than two times *N* (%)		Total before	Total during
		Before	During	Before	During	Before	During		
Age group (years)									
< 45 years old	347 (86.5)	8 (2)	28 (7)	1 (0.2)	7 (1.7)	3 (0.7)	2 (0.5)	12 (3)	37 (9.2)
> 45 years old	54 (13.5)	1 (0.2)	2 (0.5)	0 (0)	1 (0.2)	0 (0)	1 (0.2)	1 (0.2)	4 (1)
Owning dogs									
Dog owners	230 (57.4)	6 (1.5)	24 (6)	1 (0.2)	6 (1.5)	2 (0.5)	2 (0.5)	9 (2.2)	32 (8)
Respondents without dogs	171 (42.6)	3 (0.7)	6 (1.5)	0 (0)	2 (0.5)	1 (0.2)	1 (0.2)	4 (1)	9 (2.2)
Residence environment									
Urban	216 (53.9)	4 (1)	10 (2.5)	0 (0)	3 (0.7)	2 (0.5)	0 (0)	6 (1.5)	13 (3.2)
Peri/suburban/rural	185 (46.1)	5 (1.2)	20 (5)	1 (0.2)	5 (1.2)	1 (0.2)	3 (0.7)	7 (1.7)	28 (7)
Amount of time spent outdoors									
Decreased	259 (64.6)	6 (1.5)	11 (2.7)	0 (0)	1 (0.2)	2 (0.5)	1 (0.2)	8 (2)	13 (3.2)
Increased	69 (17.2)	2 (0.5)	10 (2.5)	1 (0.2)	6 (1.5)	0 (0)	1 (0.2)	3 (0.7)	17 (4.2)
No change	73 (18.2)	1 (0.2)	9 (2.2)	0 (0)	1 (0.2)	1 (0.2)	1 (0.2)	2 (0.5)	11 (2.7)
Total	401 (100)	9 (2.2)	30 (7.5)	1 (0.2)	8 (2)	3 (0.7)	3 (0.7)	13 (3.2)	41 (10.2)

Before: 1–17 March 2020; During: 18 March–15 May 2020; Total before/Total during: reports of respondents finding ticks at least one time. No reports of finding ticks were submitted by respondents over 74 years old

**Table. 3:** OLS regression analysis for selected variables

Variables	Estimate (unstandardized)	SE (standard error)	95% CI	*P*-value
Intercept	0.075	0.095	[−0.113; 0.262]	0.434
Gender (male)	−0.044	0.050	[−0.142; 0.054]	0.380
Age (under 45)	−0.011	0.065	[−0.138; 0.117]	0.870
Change in residence	−0.068	0.079	[−0.223; 0.088]	0.392
Residence type	−0.052	0.049	[−0.149; 0.044]	0.285
Owning a dog	0.076	0.048	[−0.019; 0.171]	0.116
Increase in time spent outside	0.207	0.076	[0.059; 0.356]	0.006**
Decrease in time spent outside	−0.023	0.065	[−0.151; 0.105]	0.728
Time spent in paved urban areas	0.016	0.020	[−0.023; 0.056]	0.418
Time spent in urban parks or gardens	−0.016	0.021	[−0.057; 0.026]	0.462
Time spent in nearby forests	0.016	0.019	[−0.021; 0.054]	0.398
Time spent hiking	0.060	0.020	[0.020; 0.100]	0.004**
Time spent in private/common gardens of the dwelling	−0.010	0.016	[−0.043; 0.022]	0.533
Time spent in agricultural areas	0.044	0.019	[0.006; 0.081]	0.022*

Baseline values are reported inside brackets. Asterisks indicate significant differences between values *P ≤ 0.05, **P ≤ 0.01, ***P ≤ 0.001

#### Frequency of tick bites reported from dogs

Dog owners also reported finding ticks more frequently on their dogs during the lockdown (*t*_(266)_ = −4.808, *P* = 0.000) (Table 4). Of the 230 dog owners, 65.6% (*n* = 151) reported finding ticks on their dogs during 2020, while three persons refrained from answering. A statistically significant effect was registered regarding the gender of dog owners (OLS regression, *B* = −0.419, *SE* = 0.177, *P* = 0.019), and the decrease in protective practices used for dogs (OLS regression, *B* = 0.910, *SE* = 0.221, *P* = 0.000) concerning the frequency of tick reports from dogs. The tested regression model was significant *F*_(12, 201)_ = 3.329, *P* = 0.000, with an *R*^2^ = 0.166 (Table 5).

**Table 4:** Frequency of finding ticks on dogs before and during the COVID-19 lockdown as reported by owners by selected socio-demographic characteristics

Variable *N* (%)		One time *N* (%)		Two times *N* (%)		More than two times *N* (%)		Total before	Total during
		Before	During	Before	During	Before	During		
Owning dogs as reported by owners by age group									
< 45 years old	204 (88.7)	25 (10.9)	33 (14.3)	7 (3.1)	17 (7.4)	17 (7.4)	31 (13.5)	49 (21.3)	81 (35.2)
> 45 years old	26 (11.3)	3 (1.3)	5 (2.2)	3 (1.3)	2 (1)	3 (1.3)	5 (2.2)	9 (3.9)	12 (5.2)
Environment for walking dogs									
Urban	108 (47)	12 (5.2)	15 (6.5)	5 (2.2)	6 (2.6)	3 (1.3)	15 (6.5)	20 (8.7)	36 (15.7)
Peri-urban/suburban/rural	122 (53)	16 (7)	23 (10)	5 (2.2)	13 (5.7)	17 (7.4)	21 (9.1)	38 (16.5)	57 (24.8)
Total	230 (100)	28 (12.2)	38 (16.5)	10 (4.4)	19 (8.3)	20 (8.7)	36 (15.7)	58 (25.2)	93 (40.4)

Before: 1–17 March 2020; During: 18 March–15 May 2020; Total before/Total during: reports of dog owners finding ticks on dogs at least one time. No reports of finding ticks were submitted by dog owners over 74 years old

**Table. 5:** OLS regression analysis for selected variables

Variables	Estimates (unstandardized)	SE (standard error)	95% CI	***P***-value
Intercept	0.883	0.288	[0.316; 1.450]	0.002**
Gender (male)	−0.419	0.177	[−0.768; −0.069]	0.019*
Age (under 45)	−0.015	0.230	[−0.468; 0.438]	0.948
Change in residence	0.402	0.254	[−0.100; 0.903]	0.116
Residence type (peri-urban/suburban/rural)	−0.166	0.168	[−0.497; 0.165]	0.324
Increase in time spent outdoors	0.018	0.230	[−0.436; 0.472]	0.938
Decrease in time spent outdoors	0.188	0.187	[−0.181; 0.558]	0.316
Decrease of protective measures for dogs	0.910	0.221	[0.474; 1.347]	^***^
Time spent in paved urban areas	−0.043	0.072	[−0.185; 0.098]	0.546
Time spent in urban parks or gardens	−0.011	0.068	[−0.147; 0.124]	0.867
Time spent in nearby forests	0.119	0.064	[−0.007; 0.246]	0.065
Time spent hiking	0.047	0.070	[−0.091; 0.185]	0.503
Time spent in private gardens	0.001	0.055	[−0.108; 0.110]	0.983

Baseline values are reported inside brackets. Asterisks indicate significant differences between values *P ≤ 0.05, **P ≤ 0.01, ***P ≤ 0.001

#### Recreational preferences of respondents

Most people (65%) reported spending less time outdoors once the movement restrictions became active, while 18% expressed no change, and 17% of respondents mentioned an increase in their outdoor time. Of the respondents who increased their outdoor time, 26% also changed their residence, 89% from urban to rural settlements respectively.

Moreover, when analysing the respondents’ preferences for leisure-time locations before the lockdown, the majority (89%) indicated spending the largest amount of time (under 1 h to over 2 h) in paved urban areas (commercial areas, paved playgrounds around blocks) and (85%) in urban parks or gardens with trees and shrub, followed by (63%) hiking in other areas outside their hometown (including visits to rural areas), (58%) walking in forests near the household or around the home town, (56%) staying in the private or common garden of the dwelling, and lastly (27%), frequenting agricultural areas (agricultural activities).

**Fig. 2:**
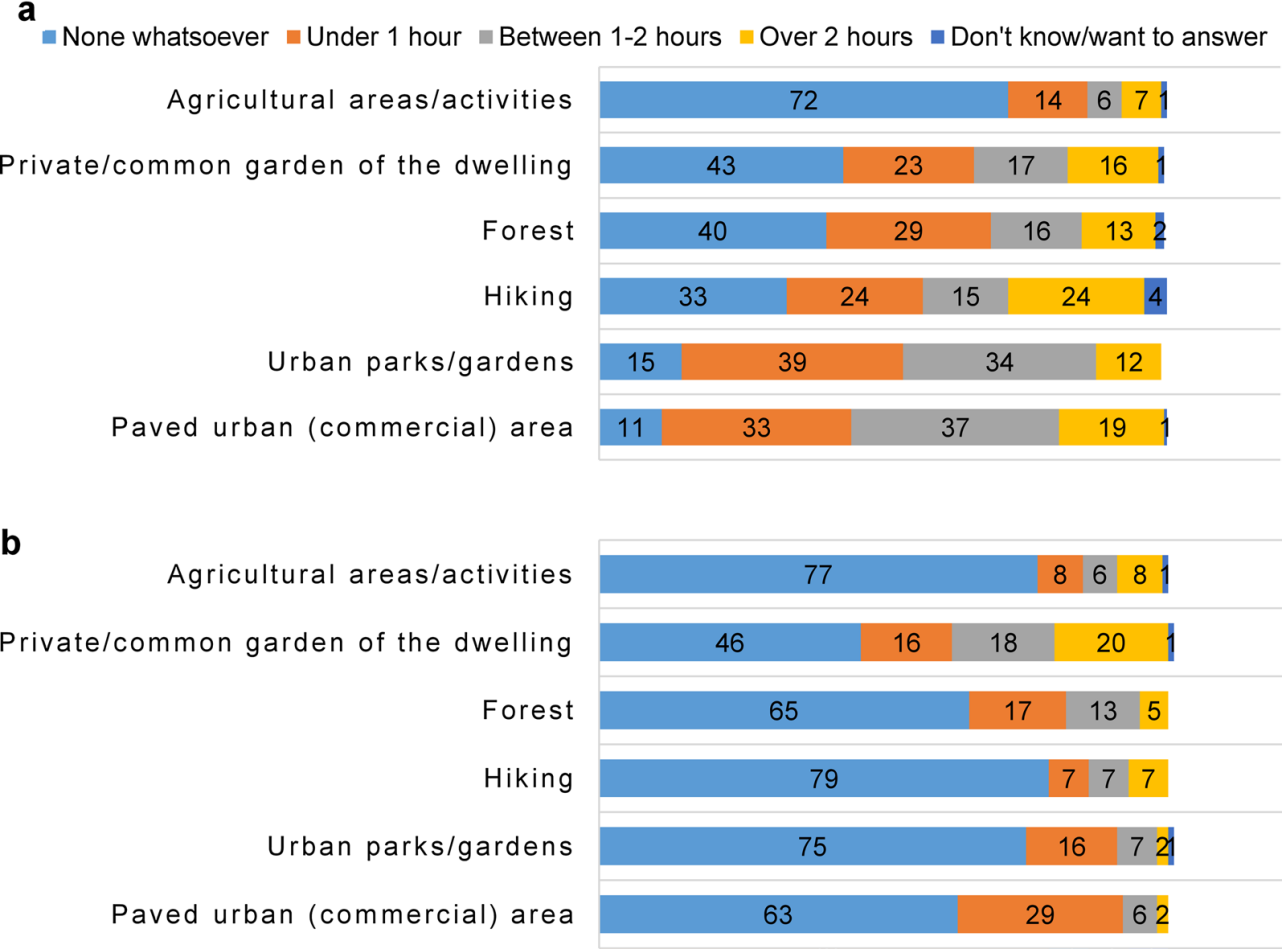
Amount of time (%) and location type respondents frequented (**a**) before and (**b**) during the COVID-19 lockdown in Romania

A decrease in the amount of time spent outdoors was noticed during the movement restrictions. The favourite recreational location of (54%) of participants (under 1 h to over 2 h) was the private or common garden of the dwelling, followed by paved urban areas (37%), forests (35%), and urban parks or gardens (25%). Finally, the least favourite recreational areas/ activities were agricultural sites (22%) and hiking (21%), respectively (Fig. 2).

Additionally, most of the children of respondents (63%) spent less time outdoors during the movement restrictions. Only (22%) stated an increase, and the rest (15%) mentioned no change in their child’s outdoor schedule.

#### Recreational behaviour of dogs described by the owners

When questioned about their dog’s outdoor schedule, the majority of owners (58%) mentioned no change, some (28%) decreased the walking duration, and a smaller share (14%) increased their dog’s outdoor time during the lockdown.

Before the movement restrictions, the majority of dog owners walked their dogs (under 1 h to over 2 h) in the private/common garden of the dwelling (67%), followed by urban parks/gardens with trees and shrubs (55%), forests near the household/around the home town (53%), paved urban areas (52%), and lastly, hiking in other areas outside their home town (including visits to rural areas) (50%).

**Fig. 3:**
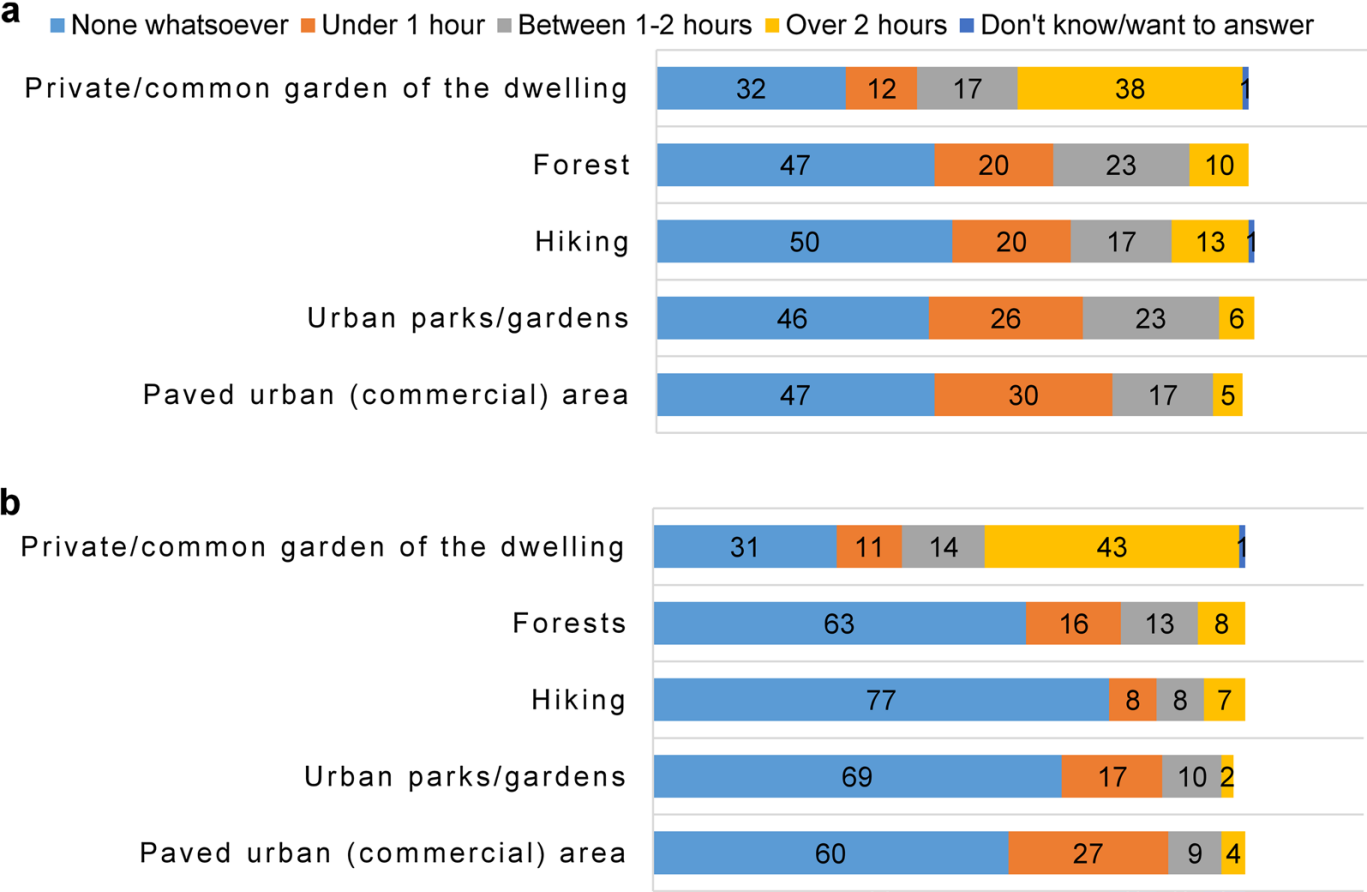
Amount of time (%) and location type where owners walked their dogs (**a**) before and (**b**) during the COVID-19 lockdown in Romania

During the lockdown, the private or common garden of the dwelling (68%) remained the first choice for dog walking, followed by paved urban areas (40%), forests (37%), urban parks/gardens (29%), and lastly hiking (23%) (Fig. 3).

### Risk perceptions

Most respondents (40%) stated that the lockdown decreased the risk of acquiring a tick or a TBD, while a substantial share (37%) responded that the lockdown did not influence the risk. Some were not sure whether the movement restrictions impacted the risk (14%), and only (8%) reported that the risk increased due to the lockdown. The perceived risk for humans was significantly correlated with the residence type (Chi-square, *χ*^2^ = 15.433, *df* = 2, *P* = 0.000) and gender (Chi-square, *χ*^2^ = 6.524, *df* = 2, *P* = 0.038), while the age group (Chi-square, *χ*^2^ = 0.107, *df* = 2, *P* = 0.948) was not a significant predictor.

When referring to their children, most respondents (41%) stated that the lockdown decreased the risk of them acquiring a tick or a TBD, followed by (33%) who felt like the lockdown did not influence the risk. Some responded with: “I don’t know” (15%), while fewer participants reported that the risk increased due to the movement restrictions (11%). The perceived risk in the case of children was significantly correlated with residence type (Chi-square, *χ*^2^ = 12.622, *df* = 2, *P* = 0.002). To test the correlation between the age group of respondents with the perceived risk for children we used the Fisher-Freeman-Halton exact test, as more than 20% of cells did not have a minimum of five cases. Neither the age (Fisher-Freeman-Halton exact test, *P* = 0.339) nor the gender (Chi-square, *χ*^2^ = 2.398, *df* = 2, *P* = 0.301) of participants were significantly correlated with perceived risk for the children in care.

Most dog owners (56%) considered that the risk their dogs were exposed to, regarding tick bites and TBDs, was not changed by the lockdown, while the rest either reported the risk had decreased (18%) or increased (14%) as a result of the movement restrictions. Some people responded with: “I don’t know” (12%). Of the tested variables, only the residence type was significantly correlated with the perceived risk of acquiring a tick or a TBD in the case of dogs (Chi-square, *χ*^2^ = 6.132, *df* = 2, *P* = 0.047). The age (Fisher-Freeman-Halton exact test, *P* = 0.335) and gender (Chi-square, *χ*^2^ = 3.846, *df* = 2, *P* = 0.146) of dog owners had no a significant correlation with the perceived risk.

### Protective practices

**Fig. 4:**
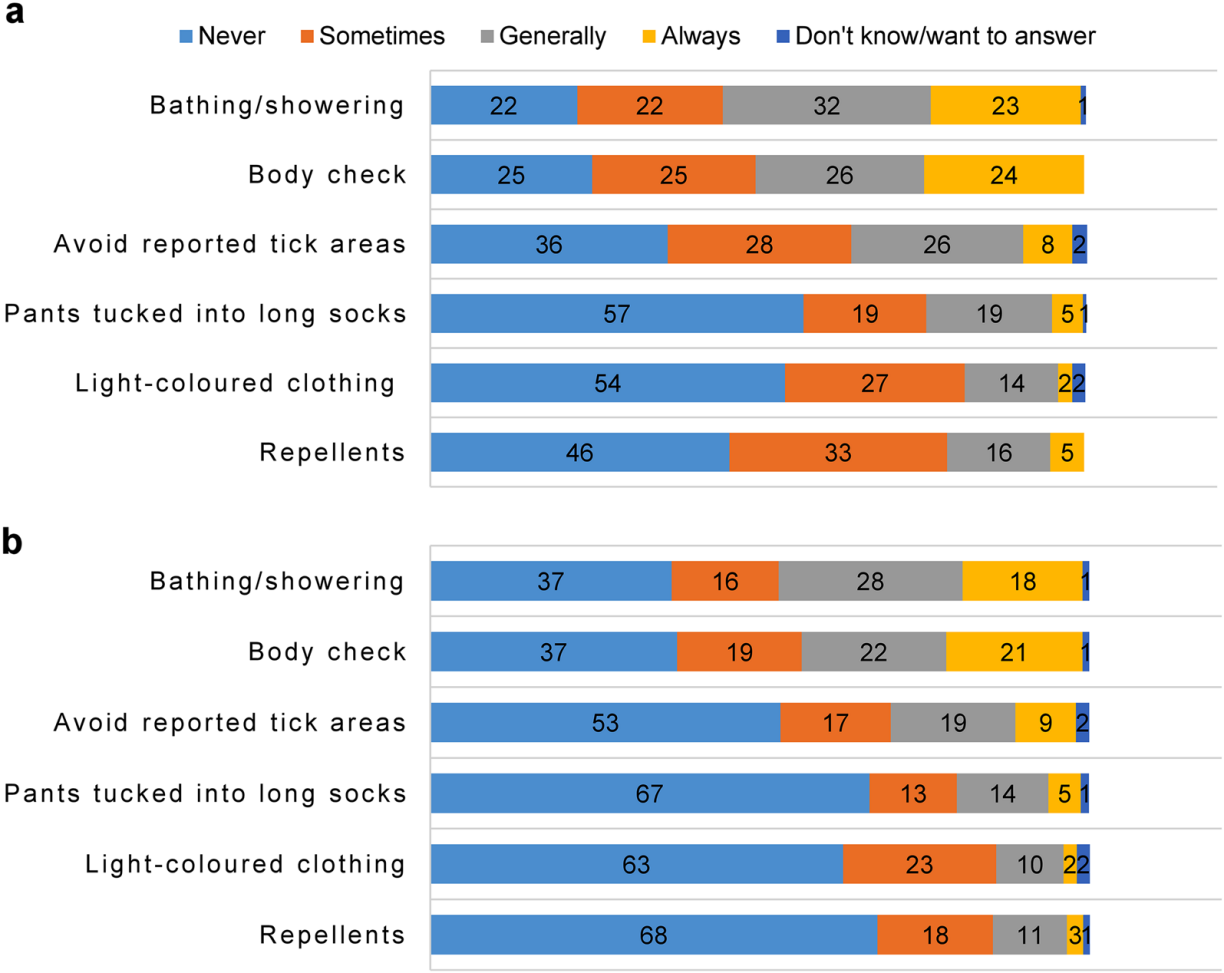
Share (%) of respondents using different protective measures against ticks (**a**) before and (**b**) during the COVID-19 lockdown in Romania

Overall, all six protection measures presented a significantly lower usage rate during the COVID-19 lockdown (Table 6). Bathing or showering, and checking the body for ticks were the two most used protective practices against ticks reported by participants both before and during the COVID-19 lockdown (Fig. 4).

**Table. 6:** Results of *t*-tests analysing the frequency of using protective methods against ticks before and during the COVID-19 lockdown in Romania

Protective methods used against ticks	Before		During		*t*-test
	*M*	SD	*M*	SD	
Commercial repellents	0.798	0.88	0.478	0.793	8.215***
Light-coloured clothing	0.641	0.913	0.507	0.764	4.198***
Pants tucked into long socks	0.709	0.935	0.568	0.912	4.015***
Avoiding reported tick areas	1.04	0.967	0.848	1.045	4.598***
Body check	1.498	1.116	1.262	1.170	5.020***
Bathing/showering to observe ticks	1.546	1.078	1.286	1.145	5.749***

M, mean; SD, standard deviation. Asterisks indicate significant differences between values ***P ≤ 0.001

Additionally, the multivariate results were significant for gender (MANOVA, Pillai’s trace = 0.040, *F*_(6, 380)_ = 2.627, *P* = 0.017) and residence type (MANOVA, Pillai’s trace = 0.059, *F*_(6, 380)_ = 3.964, *P* = 0.001), but not for the age group (MANOVA, Pillai’s trace = 0.022, *F*_(6, 380)_ = 1.417, *P* = 0.207). We only tested the main effects of these variables, as we were not interested in interaction effects. These results indicate that there are differences concerning the protective measures adopted during the lockdown based on gender and residence type, with females and residents of peri-urban/suburban/rural areas using protective measures against ticks with a higher frequency. These differences are significant for using commercial repellents and avoiding reported tick areas in the case of female respondents, while residents living in suburban/peri-urban/rural areas tend to use more commercial repellents, light-coloured clothing, and pants tucked into long socks as a means of protection against ticks compared to urban inhabitants (Tables 7, 8).

**Table. 7:** Multivariate analysis results regarding the frequency of using protective measures against ticks before and during the COVID-19 lockdown in Romanian respondents. Independent variable: gender

Dependent variable	*F*-value	Df	df error	*P*-value	Gender	Means (SE)	99% CI
Commercial repellents	4.626	1	385	0.032	Male	0.277 (0.085)	[0.109; 0.444]
					Female	0.469 (0.064)	[0.342; 0.596]
Light-coloured clothing	0.093	1	385	0.760	Male	0.474 (0.084)	[0.310; 0.638]
					Female	0.501 (0.063)	[0.376; 0.625]
Pants tucked into long socks	0.247	1	385	0.620	Male	0.468 (0.096)	[0.278; 0.657]
					Female	0.518 (0.073)	[0.374; 0.661]
Avoiding reported tick areas	4.507	1	385	0.034	Male	0.522 (0.113)	[0.300; 0.745]
					Female	0.775 (0.086)	[0.607; 0.944]
Body check	2.445	1	385	0.119	Male	0.988 (0.128)	[0.737; 1.239]
					Female	1.198 (0.097)	[1.008; 1.388]
Bathing/showering to observe ticks	0.199	1	385	0.656	Male	1.249 (0.126)	[1.001; 1.496]
					Female	1.190 (0.095)	[1.002; 1.377]

**Table. 8:** Multivariate analysis results regarding the frequency of using protective measures against ticks before and during the COVID-19 lockdown in Romanian respondents. Independent variable: residence type

Dependent variable	*F*-value	Df	df error	*P*-value	Residence type	Means	99% CI
Commercial repellents	11.303	1	385	0.001	Peri-urban/suburban/rural	0.506 (0.076)	[4.626; 0.656]
					Urban	0.239 (0.069)	[0.093; 0.374]
Light-coloured clothing	3.929	1	385	0.048	Peri-urban/suburban/rural	0.565 (0.075)	[0.247; 0.712]
					Urban	0.410 (0.067)	[4.507; 0.543]
Pants tucked into long socks	14.215	1	385	0.000	Peri-urban/suburban/rural	0.662 (0.087)	[2.445; 0.833]
					Urban	0.323 (0.078)	[0.199; 0.476]
Avoiding reported tick areas	0.059	1	385	0.808	Peri-urban/suburban/rural	0.662 (0.102)	[0.462; 0.862]
					Urban	0.636 (0.091)	[0.456; 0.816]
Body check	1.799	1	385	0.181	Peri-urban/suburban/rural	1.173 (0.115)	[0.948; 1.398]
					Urban	1.013 (0.103)	[0.811; 1.216]
Bathing/showering to observe ticks	0.398	1	385	0.529	Peri-urban/suburban/rural	1.256 (0.113)	[1.034; 1.479]
					Urban	1.182 (0.102)	[0.982; 1.382]

Despite the lack of significant correlation between the age group of respondents and the protective measures used against ticks, participants < 45 years old performed body checks and took baths/showers to observe ticks more often than people > 45 years old. Also, respondents aged > 45 chose to tuck the pants into long socks more frequently compared to younger participants.

#### Protective practices used for dogs

**Fig. 5:**
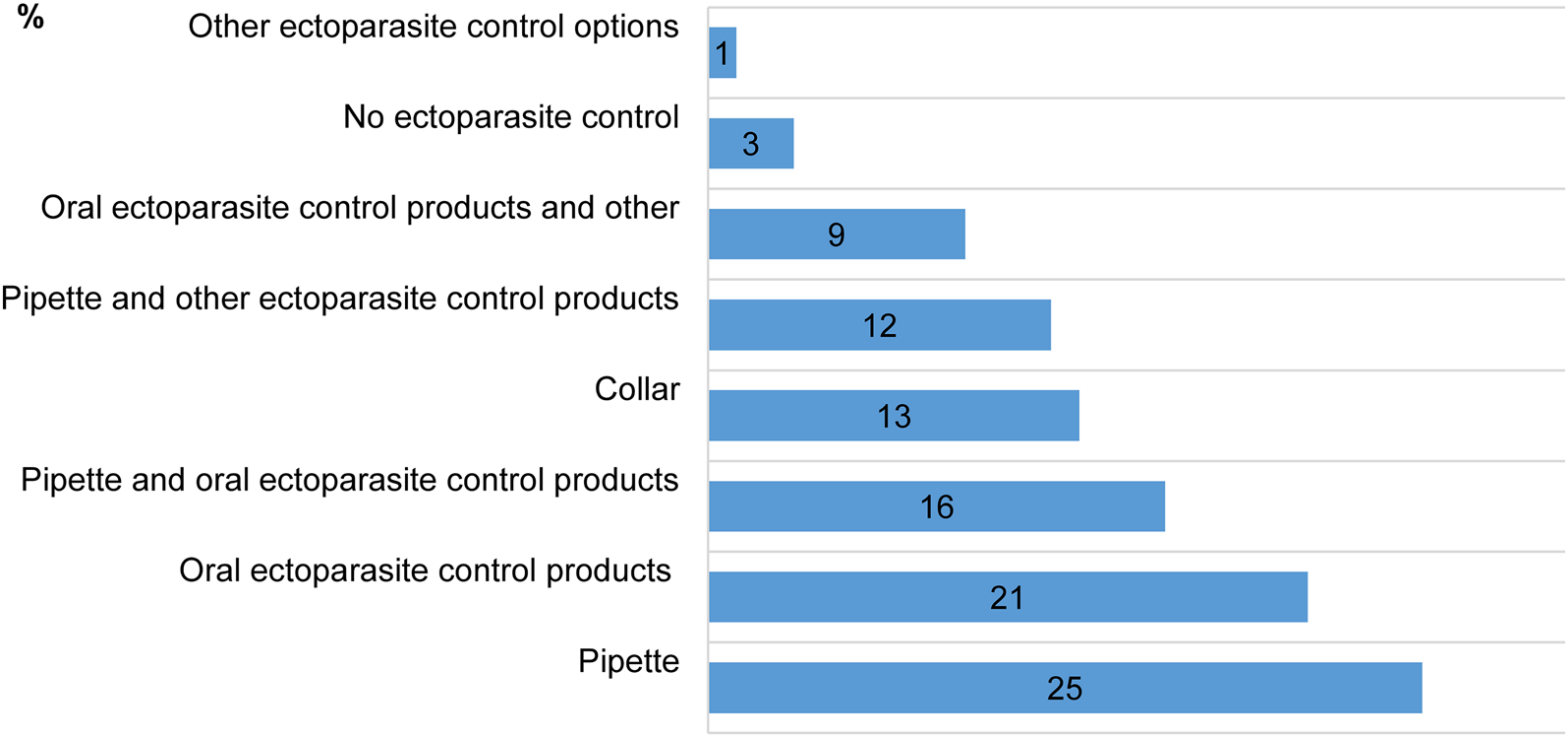
Ectoparasite control methods (%) used by owners for their dogs. Other ectoparasite control options include sprays and essential oils

Concerning the ectoparasite control methods used by the dog owners, the top three choices were the pipette (spot-on treatment) (25%), followed by products with oral administration (21%), and a combination of products with oral administration and pipette (16%). Seven (3%) respondents mentioned not performing ectoparasite control for their dogs (Fig. 5). Only a few dog owners changed their approach regarding the frequency of ectoparasite control (12%). Of these, some (4%) started using external antiparasitic/repellent products although before the restrictions they had not used them, others (4%) increased the frequency of or (1%) started using supplementary methods, and a few (1%) reduced the frequency. Some owners (1%) stopped using external antiparasitic/repellent products, due to the veterinary practice being closed (*n* = 1), considering the risk of tick bites is lower than before the movement restrictions (*n* = 1), and as a means to reduce or avoid traveling to public spaces like veterinary clinics, pharmacies, and pet shops (*n* = 1). The rest of the owners (84%) reported no change in their approach of ectoparasite control for their dogs, and (4%) chose not to respond. The decrease in the frequency of using ectoparasite control significantly increased the frequency of owners finding ticks on their dogs (OLS regression, *B* = 0.910, *SE* = 0.221, *P* = 0.000). Of the seven respondents who mentioned not performing any form of ectoparasite control on their dogs, two reported finding ticks on their dogs.

## Discussion

Since being declared an international public health emergency on 30 January 2020 by WHO, the novel SARS-CoV-2 has rapidly spread worldwide, affecting many countries in Europe, Romania included, with a still currently growing burden of disease [21]. Many countries implemented early quarantine measures as a means to control the spreading of the virus [22]. Nationwide lockdown programs have challenged the everyday life and well-being of the human population, with people worldwide experiencing various psychosocial sufferings [23]. Our goal was to assess the impact of the COVID-19 lockdown in Romania on the recreational behaviour, risk perceptions, and protective practices of the population regarding ticks.

### Recreational behaviour

As expected, most respondents in the current sample reported spending less time outdoors once the lockdown started. The same was reported by caretakers for children under 18 years old. Nevertheless, more people reported finding ticks on themselves during the lockdown. Additionally, respondents who increased their outdoor time during the lockdown reported a significantly higher frequency of finding ticks on themselves. This could be explained by yet another lifestyle adjustment induced by the lockdown, respectively a switch in the recreational location preferences of the population. Whereas previously the respondents spent the largest amount of free time in urban paved areas such as commercial areas or paved playgrounds, followed by urban parks and gardens, the restrictions forced many to find safer alternatives (sites less likely to be crowded). Therefore, the largest share of respondents chose the common or private garden of the dwelling and forests around the household for recreational purposes during the lockdown in Romania. Consequently, more people were exposed to habitats where ticks generally find suitable developmental conditions and are thus abundant [1, 24]. Also, hiking or performing agricultural activities were associated with a higher frequency of tick reports from humans. *Ixodes ricinus,* the most widespread tick species in Europe is primarily found in forested areas with shrubbery and a variety of animal hosts [1]. Thus, people who frequently visit such environments could be more exposed to ticks, and consequently to several associated TBPs, many with zoonotic potential.

The majority of dog owners mentioned no changes or a decrease in the time they spend outdoors walking their dogs. Nonetheless, they also found ticks more frequently on their dogs during the lockdown. Since the favourite location of owners for dog walking remained the same, the higher frequency of ticks reported from dogs during the lockdown could be linked to an increase in the actual amount of time dogs spent in the private/common garden of the dwelling (43% vs 38% of owners mentioned their dogs spending over 2 h in such locations). Nevertheless, our questionnaire did not assess the vegetation diversity or its management, and the fauna variety in these reported locations as to unequivocally state their suitability for tick development.

The majority of participants from a UK study that analysed the human–animal relationship during the COVID-19 lockdown declared that their pets were important emotional support and coping mechanisms during the pandemic context [25]. Moreover, nearly 70% of dogs included in another UK study were reported to have spent more time with adults during the first phase of lockdown [26]. Thus, another plausible explanation is that owners were forced to spend more time than usual at home during the lockdown in the close company of their pets, and therefore were more prone to observing changes in their dogs, such as the presence of ticks.

Nonetheless, apart from social or behavioural determinants, it is also important to mention the impact of the seasonal activity of ticks on the frequency of tick reports. Similar to information reported from Europe [1], *I. ricinus* is the most common questing tick species collected from a variety of habitats in Romania too [24, 27]. Moreover, previous studies from Romania [27], and neighbouring Hungary [28] have highlighted the bimodal activity pattern of this tick species, with adults and nymphs displaying two activity peaks (one in spring and one during autumn) with a marked dominance of spring activity (April–June). Therefore, the increasing tick density over the spring months might have also played a role in the higher frequency of ticks reported by respondents during the lockdown in the herein study.

### Risk perceptions

Being fairly new to many people, with serious potential health impacts, and difficult to manage, the risks associated with exposure to ticks and TBPs make them difficult for laypeople to assess. Previous studies have thus shown that laypeople have higher risk perceptions than experts regarding ticks and TBDs [15, 29]. Nevertheless, in the current study despite more people finding ticks on themselves during the lockdown, the majority of respondents felt like the risk of encountering ticks and possibly acquiring a TBD in their case or for the children in their care decreased or did not change due to the restrictions. The majority of dog owners stated that the movement restrictions did not impact or have decreased the risks their dogs face regarding ticks and TBDs.

It is possible that the respondents felt like the chances of encountering ticks decreased due to their everyday movement being restricted, but failed to also consider the changes regarding the preferences for recreational locations. Also, in the context of the COVID-19 pandemic where the media may have intensified the perception of risks related to the SARS-CoV-2 by sometimes offering vague or misleading information [23], the risk of encountering ticks or acquiring a TBD might have been deemed as less important or of least concern by most respondents.

Significantly more female participants considered that the risk of encountering ticks increased during the lockdown. Other studies from Europe report females to be positively associated with a higher risk perception concerning tick bites [15, 30]. Gender discrepancies in risk perceptions have been previously explored by risk perception literature. Gustafsod [31] mentions differences in social roles, daily activities, and the structural power relations established between women and men based on living conditions, as a possible explanation for the risk perception disparities. Since the perceived, and not the objective, risk determines future behaviour [12, 14], in the context of ticks and TBDs the perceived risk could influence the protective behaviour [30, 32]. Thus, targeting the Romanian male population in future awareness campaigns could prove beneficial.

### Protective practices

Overall, a higher share of respondents chose not to use protective measures against ticks during the lockdown, possibly associating the decrease in outdoor time with a smaller chance of encountering ticks. The decrease in using protective measures against ticks may have also contributed to the higher frequency of tick reports during the lockdown. Respondents might have believed that their dogs were more likely than they were to encounter ticks while outside, as mentioned by Niesobecki et al. [33], thus almost all owners used a form of ectoparasite control for their dogs, and only three persons stopped during the quarantine due to various reasons.

Almost half of the participants reported never using repellents before the lockdown, and more than half of the respondents never used repellents during the restrictions. Similar results describing low rates of using repellents were reported from other European countries [15, 34], or the USA [35]. Disliking the idea of applying a chemical to their or their children’s bodies, perceiving repellents as unsafe, and the frequency or method of application of tick repellents [33] could all be possible reasons why some Romanian respondents avoid using such products.

Despite the European Centre for Disease Prevention and Control [36] recommending tucking the pants into long socks as a means to avoid tick bites, such behaviours are less likely to be performed due to being perceived as too troublesome or socially unacceptable [37–39]. Thus, it was not unexpected that this was the least used tick protection measure both before and during the lockdown, with more than half of the Romanian participants never performing this activity. Respondents with ages over 55 years old were more likely to use this method compared to participants under this age. Similar results were reported by Jepsen et al. [34] who mentioned style reasons (the preference of younger generations to dress differently and expose their ankles) as a possible explanation. Arguably, compared to the perceived barriers of using tick protection methods such as pants tucked into long socks, wearing light-coloured clothing, or using tick repellents, most participants might have believed that the perceived benefits of such prevention behaviours would not lower the likelihood, or severity of the health risk, as detailed in the health belief model (HBM) conceptual framework [40]. More respondents mentioned avoiding reported tick areas than using light-coloured clothing as protection against tick bites in the current study. Even though no nationwide tick-reporting system exists in Romania, online free applications such as VectExcel (which displays the geographical distribution of arthropod vectors, shows probability maps for the presence of the most common tick species in Romania, and offers general information regarding TBDs and tick bites), social media, and word-of-mouth are some of the instruments which help keep the public informed on tick “hot-spots” throughout the country.

Checking the body for ticks is considered the most effective behaviour to prevent Lyme disease [41, 42]. Bathing or showering after spending time outdoors to allow tick observation followed by performing body checks were the most used protection methods against ticks both before and during the lockdown in the hereby research. These results are in line with other reports which observed the use of protective practices among people from Europe [34] or the USA [43]. People under 45 years old used these two methods more frequently than respondents over 45 years old, as observed by Jepsen et al. [34]. Possible reasons for these disparities might include mobility issues or needing glasses to see ticks for the older individuals. Nevertheless, since people over 45 years old represent the highest risk group for LB apart from children [44], better efforts should be made in Romania to target risk groups using alternative ways (apart from social media) of dispensing information (i.e. TV commercials, newspapers, leaflets, regional general practitioners).

It is important to highlight the limitations of this study. We assessed protective practices against ticks by self-reporting, which may be subjected to reporting and recall bias. Also, it is impossible to determine whether these behaviours were performed correctly due to a lack of direct observation. Given that respondents were part of a convenience sample, combined with the limited time (10 days) the questionnaire was available online, make the herein study results difficult to be generalized. Most adult respondents were less than 45 years of age, and the means of dispensing the questionnaire by using social media excluded data from some of the population at high risk, including inhabitants from rural areas in Romania. Also by choosing not to repeat the questions regarding the protective practices for children we cannot certainly affirm that the same results from adults are applicable for children when it comes to protective behaviour.

## Conclusions

This study aimed to identify how the lockdown enforced by the COVID-19 pandemic in Romania impacted the recreational behaviour, risk perceptions, and protective practices of the Romanian population regarding ticks. During the lockdown, the majority of the study participants reported spending less time outside the household, a change that was also applied to the outdoor schedule of their children or owned dogs. Nonetheless, the participants reported a higher frequency of finding ticks on themselves and their dogs after the movement restrictions were enforced. Based on this survey, this outcome might have been linked to more people choosing to spend time in the private or common garden of the dwelling, and in nearby forests, or in the case of dogs, increasing the amount of time spent in such environments. An additional factor that might have contributed was the overall lower usage rate of protective measures against ticks during the lockdown. Respondent characteristics that were found to be positively associated with risk perceptions were being female and living in peri-urban/suburban/rural environments. Men of all ages and people over 45 years old should be considered specific target groups by the relevant Romanian authorities when promoting local or nationwide awareness campaigns on ticks and TBDs. Greater efforts are also needed to encourage the use of prevention behaviours. Disseminating prevention information through social media appears to be more effective for the younger public, but it excludes an important share of the Romanian public (senior citizens and inhabitants of rural areas) while also making it difficult for internet users to sometimes find scientifically sound information provided by credible health sites. Thus, sharing information through alternative channels like primary healthcare professionals and TV advertisements could help to close this gap. Further research is also required concerning the Romanian public’s willingness to practice preventive behaviours and the factors which might influence these personal choices.

## Supplementary Information

**Additional file 1: Text S1.** The English translation of the web-based questionnaire. The survey was available online for Romanian respondents from 6 May until 15 May 2020.

**Additional file 2: Dataset S1.** Romanian respondents’ answers to the web-based survey: "Questionnaire on the risk of exposure to ticks in the context of the COVID-19 pandemic”.

## Data Availability

All data generated during this study are included in this published article and its additional files. The questionnaire in Romanian is available online at: https://form.jotform.com/201085583630048?fbclid=IwAR3JSV2KTTbdkXSHukNwFJWisG6TewGoRDU6I3sZfYpU4M6p8obQfG9E4O4. An English translation of the questionnaire is included in the additional files.

## Abbreviations

LB: Lyme borreliosis
TBEV: Tick-borne encephalitis virus
TBPs: Tick-borne pathogens
TBDs: Tick-borne diseases
COVID-19: Coronavirus disease 2019
SARS-CoV-2: Severe acute respiratory syndrome coronavirus 2
WHO: World Health Organization
HBM: Health belief model
