# Supplementary material for: Recreational behaviour, risk perceptions, and protective practices against ticks: a cross-sectional comparative study before and during the lockdown enforced by the COVID-19 pandemic in Romania
Source: Parasit Vectors. 2021 Aug 23;14:423. doi: 10.1186/s13071-021-04944-7 (PMC8381130; doi:10.1186/s13071-021-04944-7)
Supplement: Supplementary file 1 — Additional file 1: Text S1. The English translation of the web-based questionnaire. The survey was available online for Romanian respondents from 6 May until 15 May 2020. [file 13071_2021_4944_MOESM1_ESM.docx]

**Additional file 1: Text S1.** The English translation of the web-based questionnaire. The survey was available online for Romanian respondents from 6 May until 15 May 2020.

The questionnaire was translated by Ph. D. student Silvia-Diana Borșan.

Questionnaire on the risk of exposure to ticks in the context of the COVID-19 pandemic

„We are Andrei Daniel MIHALCA, university professor, and Silvia-Diana BORŞAN, Ph. D. student from the Department of Parasitology and Parasitic Diseases of the University of Agricultural Sciences and Veterinary Medicine Cluj-Napoca. We invite you to take part in a study entitled „The impact of the COVID-19 pandemic in the epidemiology of ticks and transmission of associated tick-borne pathogens to humans and animals.” The purpose of this study is to determine whether the lockdown imposed on society in the context of the COVID-19 pandemic has changed the manner and duration of time that humans, or their dogs, spend outdoors and more so, whether this change is associated with a low or increased risk of exposure to ticks and tick-borne pathogens. Only persons aged 18 and above, residents of Romania when completing the questionnaire are eligible for participation. This questionnaire is anonymous and the data collected will be used for statistical purposes, most likely followed by a scientific publication. Your answers will further help formulate prevention strategies to reduce the risk of tick bites. For further questions related to this study, please contact us via e-mail: amihalca@usamvcluj.ro or silvia.borsan@usamvcluj.ro. Thank you for participating!

1. [sYear_ –CATEGORICAL–single–Must answer]

<i> Please fill in your age group </i>

- 18-24
- 25-34
- 35-44
- 45-54
- 55-64
- 65-74
- 75-84
- 85 and above
- I choose not to respond

2. [sGender_-CATEGORICAL-single-Must answer]

- M
- F
- I choose not to respond

3. [sZip/Address_–STRING-single-Must answer]

<i> Fill in the postal code (preferably) or address (locality) of the place of residence. </i>

[q1_–CATEGORICAL–single-Must answer]

Which option best describes the type of your residence?

- (_1) Apartment in an urban area
- (_2) Apartment in peri/suburban area (areas bordering cities) surrounded by forest/trees or shrubs with private/common garden
- (_3) Apartment in peri/suburban area (areas bordering cities) surrounded by a small number of trees and shrubs/no garden
- (_4) House in an urban area with a small number of trees/shrub in the area
- (_5) Suburban house with private/shared garden
- (_6) Suburban house with no garden but access to forest
- (_7) House in a rural area
- (_8) Farm
- (_9) ____ Other

[q2_–CATEGORICAL–single-Must answer]

Have you changed your residence as a result of the movement restrictions imposed in the context of the COVID-19 pandemic?

- (_1) Yes
- (_2) No

*If q2= {_1} Then q3 and q4]*

[q3_-CATEGORICAL–single-Must answer]

Please insert the postal code (preferably) or residence address used during the movement restrictions imposed by the COVID-19 pandemic.

[q4_-CATEGORICAL–single-Must answer]

Which option best describes the type of your residence during the movement restrictions imposed in the context of the COVID-19 pandemic?

- (_1) Apartment in an urban area
- (_2) Apartment in peri/suburban area (areas bordering cities) surrounded by forest/trees or shrubs with private/common garden
- (_3) Apartment in peri/suburban area (areas bordering cities) surrounded by a small number of trees and shrubs/no garden
- (_4) House in an urban area with a small number of trees/shrub in the area
- (_5) Suburban house with private/shared garden
- (_6) Suburban house with no garden but access to forest
- (_7) House in a rural area
- (_8) Farm
- (_9) ____ Other

[q5_–CATEGORICAL–single-Must answer]

Has the amount of time you spend outdoors changed since the movement restrictions generated by the COVID-19 pandemic were imposed?

- (_1) No change
- (_2) I spend more time outdoors
- (_3) I spend less time outdoors

[q6_–CATEGORICAL–single-Must answer]

Do you care for/share the household with a child/children under 18 years old?

- (_1) Yes
- (_2) No

*[If q6= {_1} Then q7 and q14]*

*[If q6= {_2} Then q8]*

[q7_-CATEGORICAL–single-Must answer]

How has the amount of time that your child/children spend outdoors changed in the context of movement restrictions imposed by the COVID-19 pandemic?

- (_1) It increased
- (_2) It decreased
- (_3) No change

[q8_-GRID–multiple-Must answer]

How much time did you spend on a daily average outdoors and in what type of locations, BEFORE the movement restrictions enforced by the COVID-19 pandemic? <i> Choose an option on each row.</i>

|  | None whatsoever | Under 1 hour | Between 1-2 hours | Over 2 hours | Don’t know/ Don’t want to answer |
| --- | --- | --- | --- | --- | --- |
| Paved urban area: shopping areas cafes, terraces, clubs malls in the city, alleys, paved playgrounds around blocks |  |  |  |  |  |
| Urban parks/gardens with trees and shrub |  |  |  |  |  |
| Forests near the household/around the hometown |  |  |  |  |  |
| Hiking in other areas outside your hometown (including visits to rural areas) |  |  |  |  |  |
| Private/common garden of the dwelling |  |  |  |  |  |
| Agricultural areas (agricultural activities) |  |  |  |  |  |

[q9_-GRID-multiple-Must answer]

How much time did you spend on a daily average outdoors and in what type of locations DURING the movement restrictions enforced by the COVID-19 pandemic? <i> Choose an option on each row.</i>

|  | None whatsoever | Under 1 hour | Between 1-2 hours | Over 2 hours | Don’t know/ Don’t want to answer |
| --- | --- | --- | --- | --- | --- |
| Paved urban area: shopping areas cafes, terraces, clubs malls in the city, alleys, paved playgrounds around blocks |  |  |  |  |  |
| Urban parks/gardens with trees and shrub |  |  |  |  |  |
| Forests near the household/around the hometown |  |  |  |  |  |
| Hiking in other areas outside your hometown (including visits to rural areas) |  |  |  |  |  |
| Private/common garden of the dwelling |  |  |  |  |  |
| Agricultural areas (agricultural activities) |  |  |  |  |  |

[q10_–GRID–multiple-Must answer]

What tick protection methods/equipment did you use when you were spending time outdoors BEFORE the movement restrictions imposed in the context of the COVID-19 pandemic? <i > Choose an option on each row.</i>

|  | Never | Sometimes | Generally | Always | Don’t know/ Don’t want to answer |
| --- | --- | --- | --- | --- | --- |
| Commercial sprays/lotions repellents for mosquitoes and ticks |  |  |  |  |  |
| Light-colored clothing to allow tick observation |  |  |  |  |  |
| Pants tucked into long socks to limit access areas for ticks |  |  |  |  |  |
| Avoiding reported tick areas |  |  |  |  |  |
| Thorough body check after spending time outdoors |  |  |  |  |  |
| Bathing/showering as soon as possible after exit to observe/detach any ticks if present |  |  |  |  |  |

[q11_–GRID–multiple-Must answer]

What tick protection methods/equipment did you use when you were spending time outdoors DURING restrictions imposed in the context of the COVID-19 pandemic? <i > Choose an option on each row.</i>

|  | Never | Sometimes | Generally | Always | Don’t know/ Don’t want to answer |
| --- | --- | --- | --- | --- | --- |
| Commercial sprays/lotions repellents for mosquitoes and ticks |  |  |  |  |  |
| Light-colored clothing to allow tick observation |  |  |  |  |  |
| Pants tucked into long socks to limit access areas for ticks |  |  |  |  |  |
| Avoiding reported tick areas |  |  |  |  |  |
| Thorough body check after spending time outdoors |  |  |  |  |  |
| Bathing/showering as soon as possible after exit to observe/detach any ticks if present |  |  |  |  |  |

[q12_–GRID–multiple-Must answer]

Did you ever find ticks on yourself? <i> Choose an option on each row.</i>

|  | Never | One time | Two times | More than two times | Don’t know/ Don’t want to answer |
| --- | --- | --- | --- | --- | --- |
| Spring 2019 |  |  |  |  |  |
| Spring 2020 - before restrictions (1-17 March) |  |  |  |  |  |
| Spring 2020 - after restrictions (18 March-present) |  |  |  |  |  |

[q13_–CATEGORICAL–single-Must answer]

Do you believe that the movement restrictions imposed by the COVID-19 pandemic have influenced the risk of acquiring a tick or tick-borne disease in your case?

- (_1) Did not influence
- (_2) Yes, the risk decreased
- (_3) Yes, the risk increased
- (_4) Don’t know

[q14_–CATEGORICAL–single-Must answer]

Do you believe that the movement restrictions imposed by the COVID-19 pandemic have influenced the risk of acquiring a tick or tick-borne disease by your child/children?

- (_1) Did not influence
- (_2) Yes, the risk decreased
- (_3) Yes, the risk increased
- (_4) Don’t know

[q15_–CATEGORICAL–single-Must answer]

Do you own a dog/dogs?

- (_1) Yes
- (_2) No

*[If q15= {_2} Then q24]*

*[If q15= {_1} Then]*

[q16_–CATEGORICAL–single-Must answer]

Since the movement restrictions imposed by the COVID-19 pandemic, the time that your dog/dogs spent outdoors:

- (_1) Remained the same
- (_2) Increased
- (_3) Decreased

[q17_–GRID–multiple-Must answer]

BEFORE the movement restrictions enforced by the COVID-19 pandemic in what areas did you walk your dog/dogs, and what amount of time did you spend on average on the daily dog walk? <i> Choose an option on each row.</i>

|  | None whatsoever | Under 1 hour | Between 1-2 hours | Over 2 hours | Don’t know/ Don’t want to answer |
| --- | --- | --- | --- | --- | --- |
| Paved urban area: commercial areas, alleys, paved playgrounds around blocks |  |  |  |  |  |
| Urban parks/gardens with trees and shrub |  |  |  |  |  |
| Forests near the household/around the hometown |  |  |  |  |  |
| Hiking in other areas outside your hometown (including visits to rural areas) |  |  |  |  |  |
| Private/common garden of the dwelling |  |  |  |  |  |

[q18_–GRID–multiple-Must answer]

SUBSEQUENT the movement restrictions enforced by the COVID-19 pandemic in what areas did you walk your dog/dogs, and what amount of time did you spend on average on the daily dog walk? <i> Choose an option on each row.</i>

|  | None whatsoever | Under 1 hour | Between 1-2 hours | Over 2 hours | Don’t know/ Don’t want to answer |
| --- | --- | --- | --- | --- | --- |
| Paved urban area: commercial areas, alleys, paved playgrounds around blocks |  |  |  |  |  |
| Urban parks/gardens with trees and shrub |  |  |  |  |  |
| Forests near the household/around the hometown |  |  |  |  |  |
| Hiking in other areas outside your hometown (including visits to rural areas) |  |  |  |  |  |
| Private/common garden of the dwelling |  |  |  |  |  |

[q19_–CATEGORICAL-multiple & string-Must answer]

What ectoparasite control methods do you use for your dog/dogs? <i>Choose all the options that apply to you.</i>

- (_1) Oral (i.e. Bravecto, Nexgard, etc.)
- (_2) Pipet (spot-on) (e.g. Advantix, Advocate, Bravecto, Frontline, Stronghold, Vectra 3D, etc.)
- (_3) Collar (e.g. Foredo, Scalibor, Kiltix, etc.)
- (_4) Spray (e.g. Frontline, Fiprex, etc.)
- (_5) I don't use ectoparasite control products for my dog
- (_6) Other

*[If q19= {_5} Then q22]*

*If q19_.Response.Value.ContainsAny({_1,_2,_3,_4,_6}) Then]*

[q20_–CATEGORICAL–single-Must answer]

Have you changed your approach regarding the frequency of ectoparasite control of your dog/dogs in the context of the movement restrictions imposed by the COVID-19 pandemic?

- (_1) Yes, I stopped using external antiparasitic/repellent products
- (_2) Yes, I reduced the frequency of ectoparasite control
- (_3) Yes, I increased the frequency of ectoparasite control
- (_4) Yes, I started using external antiparasitic/repellent products, although before the restrictions I did not use
- (_5) No, I haven't changed my approach to the frequency of ectoparasite control
- (_6) Other

*If q20_.Response.Value={_1} Then]*

[q21_–CATEGORICAL–multiple-Must answer]

Please select from the list below the reasons that made you decide to stop using external antiparasitic/repellent products for your dog/dogs after the movement restrictions imposed by the COVID-19 pandemic became active.

- (_1) Lack of funds to buy the products
- (_2) The veterinary practice you normally go to was closed/unavailable for these facilities
- (_3) The insufficient online stock of the product you wanted, or delayed delivery
- (_4) Trying to reduce/avoid traveling to public spaces/locations like veterinary clinics, pharmacies, and pet-shops
- (_5) I believe that the risk of tick bites for my dog/dogs is lower than before the movement restrictions
- (_6) Other

[q22_–GRID–multiple-Must answer]

Did you ever find ticks on your dog/dogs? <i> Choose an option on each row.</i>

|  | Never | One time | Two times | More than two times | Don’t know/ Don’t want to answer |
| --- | --- | --- | --- | --- | --- |
| Spring 2019 |  |  |  |  |  |
| Spring 2020 - before restrictions (1-17 March) |  |  |  |  |  |
| Spring 2020 - after restrictions (18 March-present) |  |  |  |  |  |

[q23_–CATEGORICAL–single-Must answer]

Do you believe that the movement restrictions imposed by the COVID-19 pandemic have influenced the risk of your dog/dogs acquiring a tick or tick-borne disease?

- (_1) Did not influence
- (_2) Yes, the risk deceased
- (_3) Yes, the risk increased
- (_4) I don’t know

[q24_–INFO–single-Optional]

As mentioned in the introduction, this is an anonymous questionnaire. However, if you want to be notified of the final results of the study, you can write below your email address to receive this information.

example@example.com
